# miR‐34a‐5p up‐regulates the IL‐1β/COX2/PGE2 inflammation pathway and induces the release of CGRP via inhibition of SIRT1 in rat trigeminal ganglion neurons

**DOI:** 10.1002/2211-5463.13027

**Published:** 2020-12-16

**Authors:** Hui Zhang, Xue‐mei Zhang, Dan‐dan Zong, Xiao‐ying Ji, Hua Jiang, Feng‐zheng Zhang, Sheng‐dong He

**Affiliations:** ^1^ The Second Affiliated Hospital of Guilin Medical University China; ^2^ College of Integrated Traditional Chinese and Western Medicine Southwest Medical University Luzhou China; ^3^ The Second Xiangya Hospital Central South University Changsha China; ^4^ The Seventh Affiliated Hospital of Sun Yat‐Sen University Shenzhen China

**Keywords:** CGRP, COX2, migraine, miR‐34a‐5p, PGE2, SIRT1

## Abstract

Migraine is a debilitating neurological condition, with a global prevalence rate of 10.68% in men and 18.79% in women. Elucidation of the molecular mechanisms underlying migraines is of great importance for improving the quality of life of patients. The release of the neuropeptide calcitonin gene‐related peptide (CGRP) from trigeminal nerve terminals is involved in the pathogenesis of migraine. Recent studies have shown that up‐regulation of miR‐34a‐5p expression is associated with acute migraine attacks. Here, we investigated whether alteration of the expression of miR‐34a‐5p induces the release of the vasoactive peptide CGRP. We isolated primary rat trigeminal ganglion neurons and performed gain‐ and loss‐of‐function assays to alter the expression level of miR‐34a‐5p. Down‐regulation of miR‐34a‐5p inhibited the expression of interleukin‐1β (IL‐1β)/cyclooxygenase 2 (COX2)/prostaglandin E2 (PGE2), decreased IL‐1β, PGE2 and CGRP release, and up‐regulated the expression of silencing information regulator 1 (SIRT1) in trigeminal ganglion, whereas overexpression of miR‐34a‐5p enhanced the expression of IL‐1β/COX2/PGE2, increased the release of IL‐1β, PGE2 and CGRP, and decreased the expression of SIRT1 in trigeminal ganglion. In addition, overexpression of miR‐34a‐5p induced apoptosis in primary rat trigeminal neurons. In summary, these findings suggest that miR‐34a‐5p up‐regulates the IL‐1β/COX2/PGE2 inflammation pathway, induces apoptosis and enhances release of CGRP via inhibition of SIRT1 expression in trigeminal ganglion neurons; thus, miR‐34a‐5p may have potential as a therapeutic target for the treatment of migraine.

## Graphical abstract

miR‐34a‐5p up‐regulates the IL‐1β/COX2/PGE2 inflammation pathway and induces apoptosis and the release of calcitonin gene‐related peptide via inhibition of SIRT1 expression in trigeminal ganglion neurons. This suggests that miR‐34a‐5p may have potential for development into a therapeutic target for the treatment of migraine.

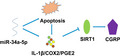

**Abbreviations**
Ac‐p65: acetylated NF‐κB p65
CGRP: calcitonin gene‐related peptide
COX2: cyclooxygenase 2
FISH: fluorescence *in situ* hybridization
IL‐1β: interleukin‐1β
lenti‐miR‐34a‐5p: lentiviral vector for overexpression of miR‐34a5p
NeuN: neuronal‐nuclear protein
NF‐κB: nuclear factor kappa B
PGE2: prostaglandin E2
SD: standard deviation
SIRT1: silencing information regulator 1

Migraine is a common, complex and disabling neurological disease with sensory changes. According to authoritative data related to migraine, the prevalence rate of men is 10.68% and the prevalence rate of women is 18.79% worldwide [1, 2]. In 2017, the annual global economic burden caused by diseases was about 853 million years lived with disability(YLDs), of which neurological diseases accounted for about 4.5% of them, and migraine accounted for >30% of the economic burden of neurological diseases [3]. Therefore, exploring the mechanism of the occurrence of migraine and finding new therapeutic targets are of great importance for improving the quality of life for patients.

The pathogenesis of migraine has yet to be studied in depth. Currently, there are mainly vascular source theory, cortical spread inhibition theory, neurovascular reflex theory, etc. Among these theories, the trigeminal neurovascular reflex theory is the dominant one [2, 4]. Trigeminal neurovascular system activation is considered to be the central link in the onset of migraine [4, 5]. The trigeminal neurovascular system is mainly composed of dura mater, intracranial artery, trigeminal ganglion, tail of trigeminal spinal tract nucleus, C1–2 dorsal nucleus of cervical spinal cord, thalamus, cortex, etc. [4]. The tail of the trigeminal spinal nucleus and the dorsal nucleus of the cervical spinal cord of C1 and C2 are collectively referred to as the trigeminal neck complex [6, 7]. When the trigeminal nerve endings on the dura mater and intracranial blood vessel wall are stimulated, it causes the trigeminal nerve unmyelinated C fibers around the blood vessel to release vasoactive substances, such as substance P, calcitonin gene‐related peptide (CGRP), neuropeptide A, etc. [7, 8, 9]. These substances can produce neurogenic inflammation, causing strong dilation of meningeal blood vessels, plasma protein exudation and mast cell degranulation. In turn, the pain response threshold of intracranial pain‐sensitive tissues, such as dura mater, pial meningeal blood vessels, and sinuses, is reduced [8, 9]. Subsequently, pain sensation passes through the trigeminal nerve ocular branch and the posterior root of C1 and C2 to the second‐order neurons of pain sensation (brain caudal trigeminal spinal nucleus caudal subnucleus), then third‐order neurons (thalamus), and finally to the cortex (front Buckle back, island back frontal cortex) and produce migraine [9].

miRNAs are a class of noncoding RNAs with posttranscriptional gene expression regulation functions *in vivo*, about 20–25 nucleotides in length, which are involved in the generation and maintenance of pain in animal models of inflammatory and neurogenic pain. Previous studies show that miRNAs may play an important role in the pathogenesis of migraine [10, 11]. Among these miRNAs, miR‐34a‐5p may be involved in the pathogenesis of migraine. Andersen *et al*. [11] found that serum miR‐34a‐5p in patients with migraine in the non‐onset period was not different from healthy people, but the expression of miR‐34a‐5p in serum was significantly increased during the attack. In addition, miR‐34a is involved in regulating interleukin‐1β (IL‐1β) expression. Overexpression of miR‐34a in cortical neurons *in vitro* can increase the expression of IL‐1β, and stress injury enhances the effect of miR‐34a on IL‐1β [12]. The protective mechanism of carbon monoxide in mouse liver ischemia/reperfusion injury participates in the down‐regulation of miR‐34a, induces the expression of deacetylase silencing information regulator 1 (SIRT1), reduces the acetylation of nuclear factor kappa B (NF‐κB) and inhibits the expressions of inflammatory factors, including IL‐1β, IL‐6 and tumor necrosis factor‐α [13]. SIRT1 directly deacetylates the P65 subunit of NF‐κB, reducing its acetylation level, thereby inhibiting the transcription function of downstream factors [14]. miR‐34a may increase the level of acetylated NF‐κB p65 (Ac‐p65) by inhibiting SIRT1 and activate IL‐1β transcription.

Prostaglandin E2 (PGE2) is the main proinflammatory prostaglandin and an important peripheral mediator of inflammation and pain [15, 16]. Cyclooxygenase 2 (COX2) can increase the synthesis of PGE2 in the central nervous system, can promote inflammatory pain and neuronal sensitization, and is related to the degree of inflammatory pain [16, 17]. When migraine triggers, pain sensitivity arises from the afferent sensory fibers of the trigeminal nerve, which extensively innervate the meningeal vessels in the skull. In the trigeminal neurovascular system, CGRP is the most abundant peptide, mainly expressed in trigeminal ganglion neurons [18]. After the trigeminal neurovascular system is activated, the release of CGRP from the trigeminal nerve terminals is an important link in the pathogenesis of migraine [5, 19]. Therefore, in this study, we aimed to investigate whether overexpressing miR‐34a‐5p could inhibit SIRT1 expression, up‐regulate the IL‐1β/COX2/PGE2 inflammatory pathway and induce the release of the vasoactive peptide CGRP in isolated rat trigeminal ganglion neurons.

## Materials and methods

### Isolation and culture of rat trigeminal ganglion neurons

For culture plate coating, add 5% Poly‐D‐lysine Solution(PDL) to the culture plate, coat 90 min at 4 °C, dry at room temperature for 5 min, wash twice with PBS; then add coating medium, coat 90 min at 37 °C, wash twice with PBS, and then add starter medium to save for future use.

All experimental procedures and animal experimental protocols were in accordance with the guidelines for laboratory animal care of the National Institutes of Health and Southwest Medical University. The experiment was approved by The Ethics Committee of the Southwest Medical University, with the license number 201703056. Efforts were made to minimize the suffering of the animals. All animals were kept in standard rooms and housed with a 12/12‐h light/dark cycle, a constant temperature (23 ± 2 °C) and 50% humidity. All animals were fed with free diet and water.

The 3‐day‐old newborn Sprague–Dawley rats were anesthetized using phenobarbital and then sacrificed with minimal suffering. The whole body of rats was soaked with 75% alcohol for 3 min and then transferred to a sterile dish on the ultraclean table with an ice box under the dish. The trigeminal ganglion of the suckling rat was isolated aseptically and then transferred to a dish containing dissociation medium and placed on ice. Sterile ophthalmic scissors were used to chop the tissue, which was transferred to 10 mL Collagenase I/Dispase digestion solution and digested at 37 °C for 90 min. After digestion, the cells were washed with precooled PBS twice, and then 10 mL papain digestion solution was added with a final concentration of 2 mg·mL^−1^; the cells were digested at 37 °C for 30 min. After the digestion, cells were centrifuged at 2100 ***g*** for 2 min, the supernatant was discarded, and the pellet was washed twice with precooled PBS and once with Dissociation medium. After resuspending the pellet in the Dissociation medium, the tissue was gently blown with a Pasteur pipette to disperse the cells. Then cells were centrifuged at 2100 ***g*** for 2 min, and the pellet was washed once with Starter medium and centrifuged at 2100 ***g*** for 2 min and then resuspended in the Starter medium. The cells were seeded into the earlier‐mentioned coated 24‐well plate according to two trigeminal ganglia per well. After 24 h of culture, the medium was changed to Neurobasal medium + B27 medium containing cytosine arabinoside to continue the culture. After 3 days, the medium was changed to Neurobasal medium + B27 medium and changed every 2 days.

### Quantitative real‐time PCR

Total RNAs were extracted from rat trigeminal ganglion cells by using TRIzol (Invitrogen,Carlsbad, CA, USA). Then RNAs were reverse transcribed into cDNA by using a reverse transcription kit (CWBio, Beijing, China). Then quantitative real‐time PCR was carried out by using the UltraSYBR mixture (CWBio). The primers for cox2, IL‐1β, NF‐κB and SIRT1 were designed by using primer 5.0 software (Premier, Vancouver, BC, Canada) after searching the target gene mRNA sequences on National Center for Biotechnology Information. The sequences for primers were listed as follows: miR‐34a‐5p, 5′‐GCGTGTCAGTGTCTTAGCTGGTTGT‐3′; β‐actin, sense 5′‐ACATCCGTAAAGACCTCTATGCC‐3′, antisense 5′‐TACTCCTGCTTGCTGATCCAC‐3′; COX2, sense5′‐AGCCATGCAGCAAATCCTT‐3′, antisense 5′‐AGTTTTCACCGTAGAATCCAGT‐3′; IL‐1β, sense 5′‐TGTGATGTTCCCATTAGAC‐3′, antisense 5′‐AATACCACTTGTTGGCTTA‐3′; SIRT1, sense 5′‐AAAGGAAATATATCCCGGACA‐3′, antisense 5′‐TTTGGATTCCTGCAACCTG‐3′; NF‐κB p65, sense 5′‐ACTATGGATTTCCTGCTTACGG‐3′, antisense 5′‐GCACAATCTCTAGGCTCGTT‐3′. β‐Actin served as the internal control to calculate the target genes relative expression by using the $2^{‐\Delta \Delta C_{t}}$ method.

### Western blot analysis

Cell lysates were prepared by radio‐immunoprecipitation assay (RIPA) buffer (ApplyGen, Beijing, China). Protein concentrations were determined by using the BCA protein assay kit (Wellbio, Changsha, China). Then an equal amount of total proteins was separated by 10% SDS/PAGE and transferred onto a nitrocellulose filter membrane (Millipore, Shanghai, China). The membranes were blocked in 5% fat‐free dry milk in TBS for 1.5 h at room temperature. Then membranes were incubated with primary antibodies against COX2 (catalog number [Cat#] 12375‐1‐AP, Rabbit, 1 : 300; Proteintech, Chicago, IL, USA), IL‐1β (Cat# ab9722, Rabbit, 0.1 µg·mL^−1^; Abcam, Cambridge，UK), NF‐κB p65 (Cat# 10745‐1‐AP, Rabbit, 1 : 1000; Proteintech), AC‐p65 (Cat# ab19870, Rabbit, 2.5 µg·mL^−1^; Abcam), SIRT1 (Cat# ab189494, Rabbit, 1 : 1000; Abcam) and β‐actin (Cat# 60008‐1‐1g, Mouse, 1 : 5000; Proteintech) for overnight at 4 °C. After being washed with cold TBST, the membranes were incubated with horseradish peroxidase‐conjugated secondary antibodies (Proteintech). At last, the specific proteins were visualized with enhanced chemiluminescence detection reagent (Thermo Fisher, Waltham, MA, USA). The intensity of protein bands was determined by the quantity one software（Bio‐rad, Richmond，California，USA ） and corrected by subtracting the measured intensity with the background intensity. The quantification was performed by dividing the intensity of each target protein with the intensity of total β‐actin on the blot.

### Fluorescence *In Situ* Hybridization

Fluorescence *in situ* hybridization (FISH) staining was performed by using FISH kit (Cat# C10910; RiboBio, Guangzhou, China). In brief, cells in the petri dish were fixed with 4% paraformaldehyde at room temperature for 30 min. Then the cells were washed with PBS three times for 5 min each time. Cells were permeabilized with 0.3% Triton X‐100 for 30 min at 37 °C. After being washed three times with PBS, cells were added with prehybridization solution at 37 °C for 30 min. Then 2.5 µL preheated 50 µM miR‐34a‐5p probes were added into the 200 µL hybridization solution to make hybridization solution, and then these solutions were added into the cells and incubated overnight in the dark. The cells were washed three times with hybridization washing solution at 42 °C for. Cells were then washed three times with PBS, and dihydrochloride (DAPI) was used for nuclear staining. The slides were then sealed with 90% glycerin and stocked in the dark for observation under a fluorescence microscope (BA410E; Motic，Xiamen，Fujian, China).

### Cell transfection and treatment

A total of 2 × 10^5^ isolated trigeminal ganglion neurons were plated into six‐well plates and cultured overnight, and then cells were cotransfected with miR‐34a‐5p inhibitor at 100 nm in the absence or presence of different types of constructs using Lipofectamine® 3000 (Thermo Fisher) for 48–72 h following the manufacturer’s instructions. Then cells were collected for indicated experiments. The sequence for miR‐34a‐5p inhibitor was 5′‐ACCGUCACAGAAUCGACCAACA‐3′.

The transfected cells were divided into seven groups for down‐regulation of miR34a‐5p and SIRT1, including Blank group, miRNA inhibitor control + siRNA control, miRNA34a‐5P inhibitor group, miRNA34a‐5p inhibitor + siRNA control group, SIRT1 siRNA group, miRNA inhibitor control + SIRT1 siRNA group and miRNA34a‐5p inhibitor + SIRT1 siRNA group. Another set of transfected cells was divided into seven groups for overexpression, including Blank group, normal culture without transfection; miRNA control + siRNA control, cotransfected with rno‐miR‐34a‐5p, overexpressing empty lentivirus and IL‐1β silent empty lentivirus; miRNA34a‐5p overexpression group, transfected with lentiviral vector for overexpression of miR34a‐5p (lenti‐mir‐34a‐5p) overexpression lentivirus; miRNA34a‐5p + siRNA control group, cotransfected with lenti‐miR‐34a‐5p overexpressed lentivirus and IL‐1β‐silenced empty lentivirus; IL‐1β siRNA group, transfected with IL‐1β‐silencing lentivirus; miRNA control + IL‐1β siRNA group, cotransfected with rno‐miR‐34a‐5p‐overexpressing empty lentivirus and IL‐1β silent lentivirus; and miRNA34a‐5p + IL‐1β siRNA group, cotransfected with rno‐miR‐34a‐5p overexpression lentivirus and IL‐1β‐silenced lentivirus.

### ELISA

The earlier‐mentioned transfected cells were collected by centrifugation at 1000 ***g*** for 5 min. After being washed with PBS, the cells were resuspended with PBS and put in the refrigerator at −20 °C overnight. Then the cells were thawed and frozen three times to lysate cells. Cell lysates were collected by centrifugation at 5000 ***g*** for 15 min, and the supernatants were used to detect the protein expression of PGE2, IL‐1β and CGRP by using the ELISA kits [IL‐1β (Proteintech, Wuhan, China) and PGE2 and CGRP (CUSABIO, Wuhan, China)] following the manufacturer’s protocols. The absorbance of each sample was detected at *OD*_450_ by Multifunctional microplate reader (MB‐530; Huisong Technology Development Co., Ltd, Shenzhen, China).

### Immunofluorescence and microscopy

The successful isolation of rat trigeminal ganglion neurons was verified by immunofluorescence. In brief, cells were fixed with 4% paraformaldehyde for 30 min at room temperature and permeabilization with 0.3% Triton X‐100 for 5 min and blocking with 10% BSA in PBS for 1 h. Cells were then stained with NeuN (bs‐1613r; Bioss, Boston, MA, USA) and β‐Tubulin III (ab18207; Abcam), for overnight at 4 °C. After washing with PBS, cells were incubated with Alexa Fluor‐549‐conjugated secondary antibodies (SA00006‐8; Proteintech, China) for 1.5 h at room temperature. Nuclei were stained with 1 mg·mL^−1^ DAPI. Images were acquired via fluorescent microscope (BA410E; Motic).

### Flow cytometry analysis

The cells were collected after washing with PBS, following the instructions of Annexin V–FITC/propidium iodide (KGA108; KeyGen Biotech, Nanjing, Jiangsu, China). Subsequently, we added binding buffer suspension, Annexin V–FITC and propidium iodide. After mixing, the cells were incubated for 15 min. Apoptosis was detected by flow cytometry (Becton, Dickinson and Company, NJ, USA).

### Statistical analysis

All the data are presented as means ± standard deviation (SD). Comparisons among multiple groups were analyzed by one‐way ANOVA, followed by Tukey’s *post hoc* test. A *P* value <0.05 was considered to be statistically significant. All experiments were repeated at least three times with similar results.

## Results

### Successful isolation and culture of rat trigeminal ganglion neurons

**Fig 1:**
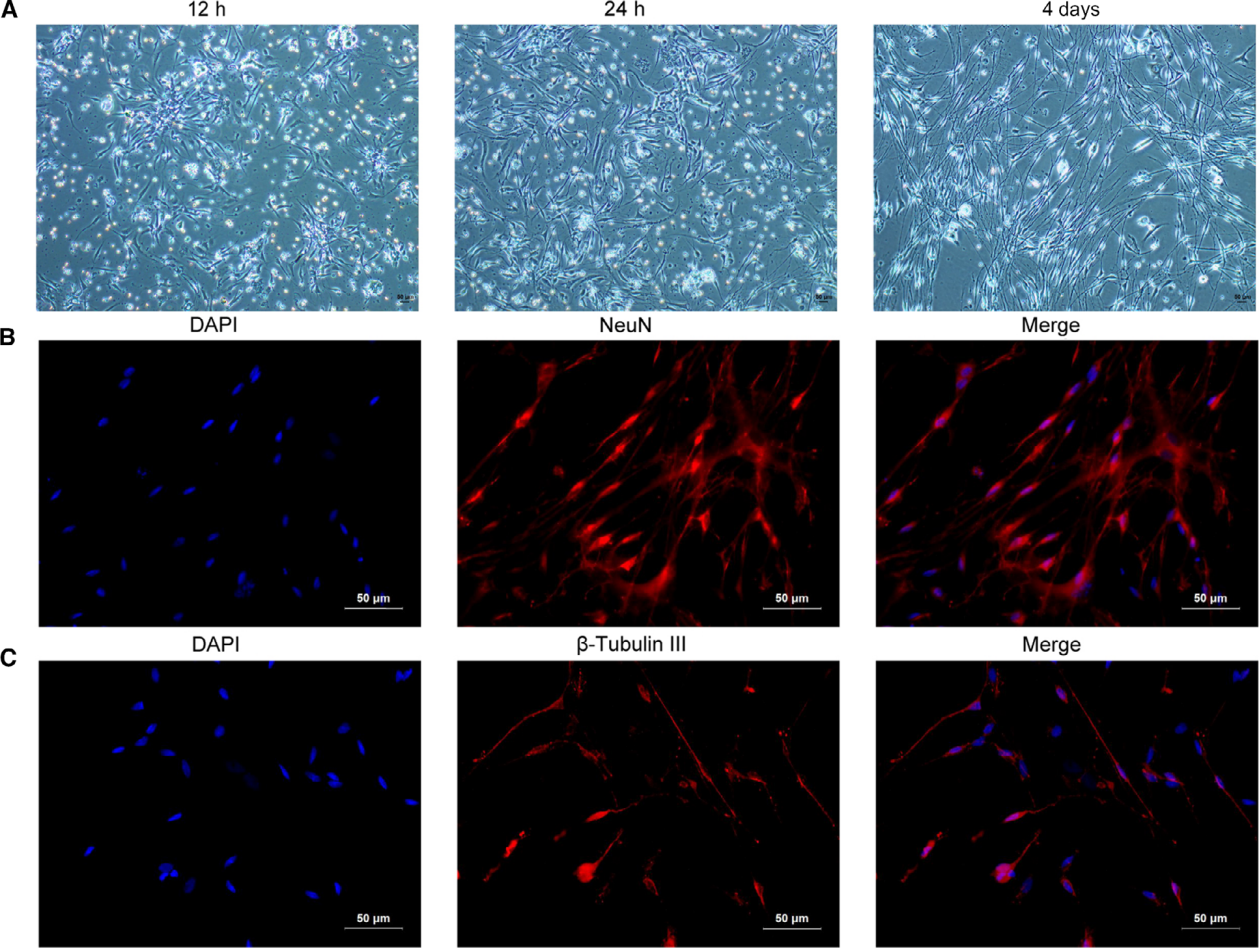
Isolation and culture of rat trigeminal ganglion neurons. (A) Isolated rat trigeminal ganglion neurons were cultured for 12 h, 24 h and 4 days, and then images were acquired by inverted microscope. (B) Isolated rat trigeminal ganglion neurons were stained with the nuclear dye DAPI (blue) and immunostained for NeuN (red). (C) Isolated rat trigeminal ganglion neurons were stained with β‐Tubulin III. Each experiment was repeated three times with similar results. Scale bars: 50 μm.

First, we isolated the rat trigeminal ganglion neurons from rats by using Collagenase I/Dispase digestion solution. After the cells were cultured for 12 h, 24 h and 4 days, we observed the trigeminal ganglion neurons growth under a microscope (Fig. 1A). To confirm the successful isolation, we stained the cells with trigeminal ganglion neurons with NeuN and β‐tubulin III. The immunofluorescence staining showed the positive staining for both of these two markers (Fig. 1B,C), indicating the successful isolation of trigeminal ganglion neurons. Therefore, the cells were used for the subsequent functional studies.

### Down‐regulation of miR‐34a‐5p inhibited the IL‐1β/COX2/PGE2 inflammation pathway via up‐regulation of SIRT1 in trigeminal ganglion neurons

**Fig 2:**
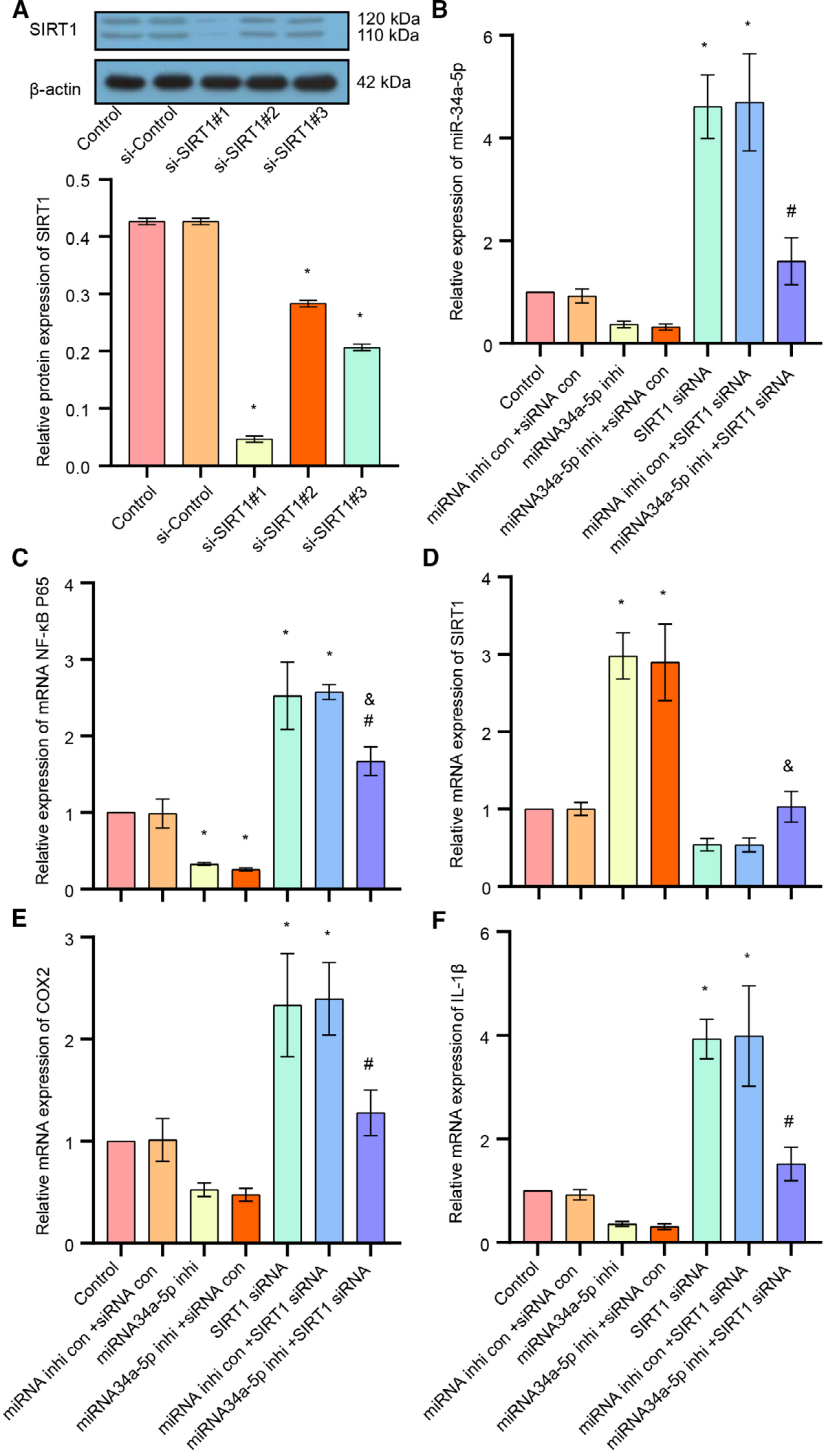
The effect of down‐regulation of miR‐34a‐5p/SIRT1 on the IL‐1β/COX2/PGE2 inflammation pathway and CGRP expression in trigeminal ganglion. (A) The expression level of SIRT1 was examined after cells transfected with si‐Control or si‐SIRT1 lentivirus, detected by western blot. (B) The expression of miR‐34a‐5p in each group was detected by quantitative real‐time PCR. (C) The expression of NF‐κB p65 in each group was detected by quantitative real‐time PCR. (D) The expression of SIRT1 in each group was detected by quantitative real‐time PCR. (E) The expression of COX2 in each group was detected by quantitative real‐time PCR. (F) The expression of IL‐1β in each group was detected by quantitative real‐time PCR. Data are shown as means ± SD. *P < 0.05, versus the control group; ^#^ *P* < 0.05, versus the miRNA inhibitor control + SIRT1 siRNA group; ^&^ *P* < 0.05, versus the miRNA34a‐5p inhibitor + siRNA control group. Each experiment was repeated three times with similar results.

We silenced the endogenous SIRT1 with or without siR‐34a‐5p inhibitor by using siRNA to detect the effect on the IL‐1β/NF‐κB p65/COX2 inflammation pathway. Western blot analysis showed that si‐SIRT1#1 had the highest efficacy on silencing of SIRT1, which was then selected for the following experiments (Fig. 2A). Because miR‐34a‐5p was shown to be up‐regulated in patients with migraine, we down‐regulated miR‐34a‐5p by using its inhibitor, and then we detected the expression level of SIRT1, IL‐1β, miR‐34a‐5p, NF‐κB p65 and COX2 by quantitative real‐time PCR. The results showed that the expression of miR‐34‐5p was decreased after the cells were treated with its inhibitor (Fig. 2B). In addition, the expressions of IL‐1β, NF‐κB p65 and COX2 were also markedly decreased upon the inhibition of miR‐34a‐5p, whereas the expression of SIRT1 was profoundly increased, indicating that miR‐34a‐5p positively regulated the IL‐1β/NF‐κB p65/COX2 inflammation pathway, but negatively regulated the expression of SIRT1 (Fig. 2B–F).

Quantitative real‐time PCR results showed that silencing of SIRT1 significantly increased the expression of IL‐1β/NF‐κB p65/COX2 and siR‐34 a‐5p (Fig. 2B–F), whereas inhibition of miR‐34a‐5p attenuated the silencing of SIRT1‐induced up‐regulation of IL‐1β/NF‐κB p65/COX2 and siR‐34a‐5p (Fig. 2B–F).

### Down‐regulation of miR‐34a‐5p inhibited the IL‐1β/COX2/PGE2 inflammation pathway and CGRP release via SIRT1 in trigeminal ganglion neurons

**Fig 3:**
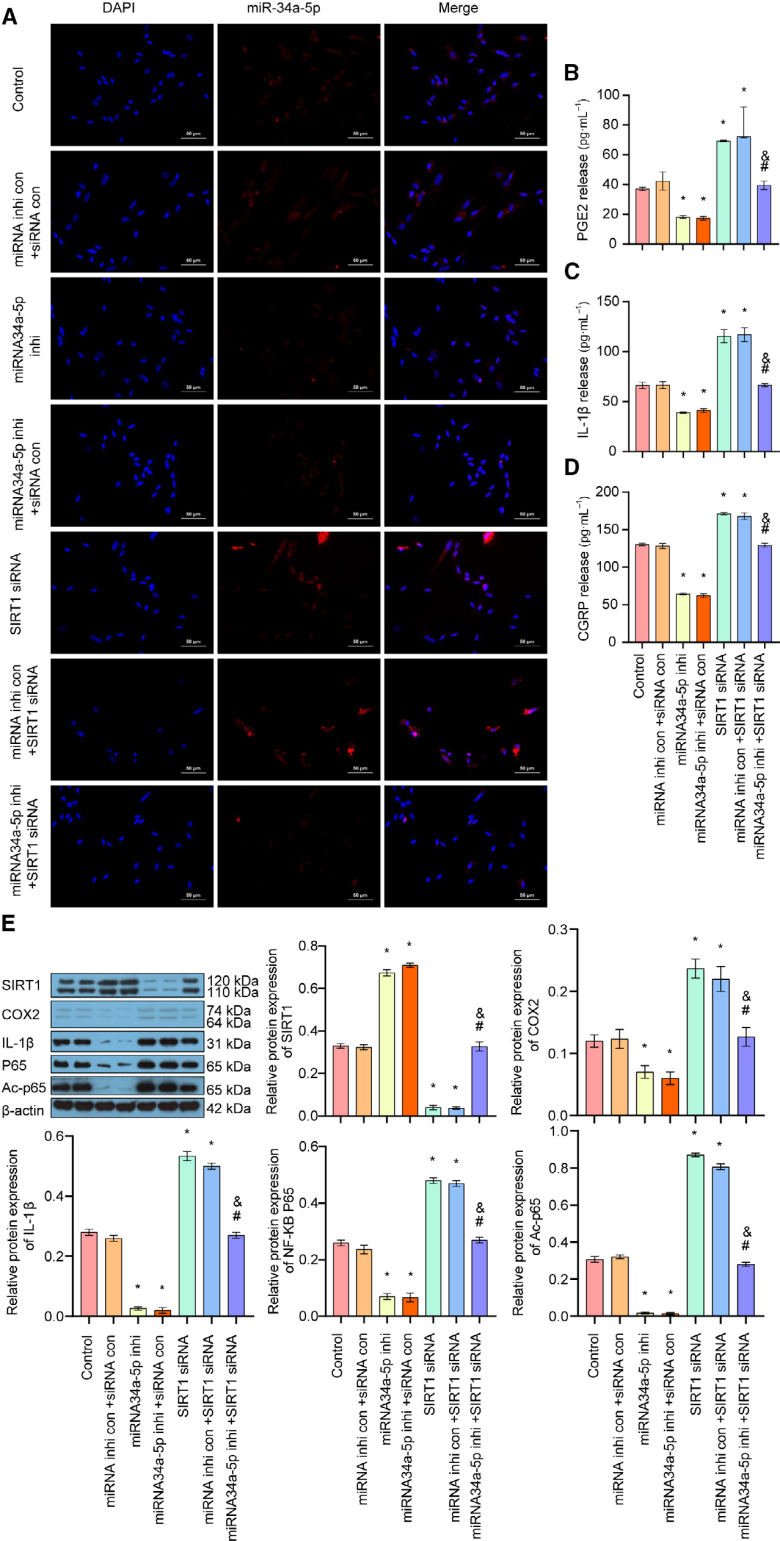
The effect of down‐regulation of miR‐34a‐5p/SIRT1 on the IL‐1β/COX2/PGE2 inflammation pathway and CGRP release in trigeminal ganglion. (A) The expression and localization of miR‐34a‐5p in each group was detected by FISH assay (scale bar: 50 μm). (B) The release of PGE2 in each group was examined by ELISA. (C) The release of IL‐1β in each group was detected by ELISA. (D) The release of CGRP in each group was detected by ELISA. (E) The expressions of SIRT1, COX2, IL‐1β, p65 and AC‐p65 in each group were detected by Western blot. Data are shown as means ± SD. *P < 0.05, versus the control group; ^#^ *P* < 0.05, versus the miRNA inhibitor control + SIRT1 siRNA group; ^&^ *P* < 0.05, versus the miRNA34a‐5p inhibitor + siRNA control group. Each experiment was repeated three times with similar results.

Then, we examined the expression of miR‐34a‐5p by FISH assay, and the results showed that miR‐34a‐5p inhibitor significantly decreased the expression of miR‐34a‐5p in cells, whereas down‐regulation of SIRT1 increased the expression of miR‐34a‐5p (Fig. 3A). We detected the release of PGE2, IL‐1β, and CGRP by ELISA. The results demonstrated that the release of PGE2, IL‐1β and CGRP was remarkably inhibited by miR‐34a‐5p inhibitor, whereas silencing of SIRT1 increased the release of PGE2, IL‐1β and CGPR. Moreover, down‐regulation of SITR1 attenuated miR‐34a‐5p inhibitor‐induced decreased release of PGE2, IL‐1β and CGPR (Fig. 3B–D). Moreover, Western blot analysis showed that the miR‐34a‐5p inhibitor significantly decreased the protein expressions of COX2, IL‐1β, NF‐κB p65 and AC‐p65, whereas silencing of SIRT1 increased the protein expression of COX2, IL‐1β, NF‐κB p65 and AC‐p65 and attenuated the miR‐34a‐5p‐induced down‐regulation of these proteins (Fig. 3E). Altogether, these results suggested that down‐regulation of miR‐34a‐5p inhibited the IL‐1β/COX2/PGE2 inflammation pathway and CGRP release in trigeminal ganglion via up‐regulation of SIRT1.

### Up‐regulation of miR‐34a‐5p enhanced the IL‐1β/COX2/PGE2 inflammation pathway and CGRP expression through down‐regulation of SIRT1 in trigeminal ganglion neurons

**Fig 4:**
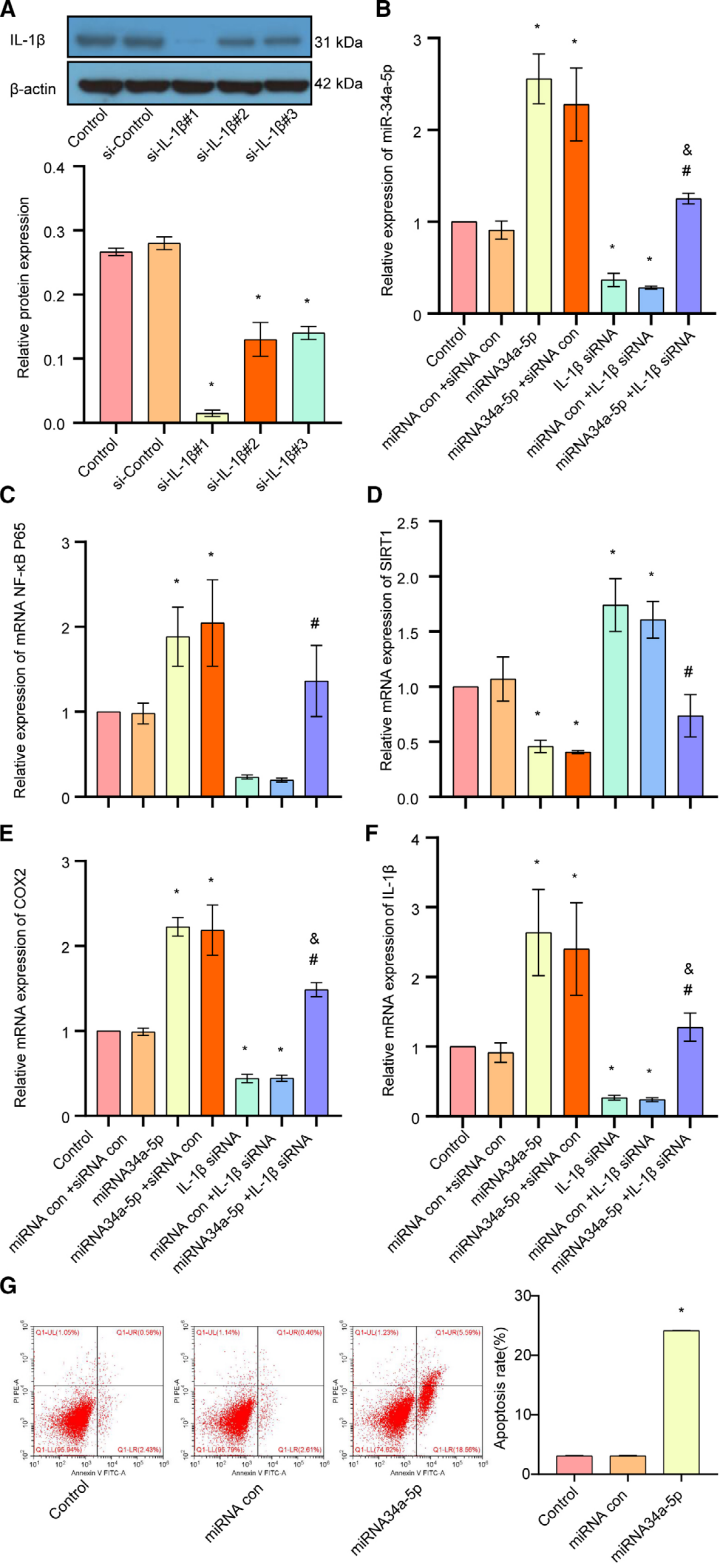
The effect of up‐regulation of miR‐34a‐5p/IL‐1β on the SIRT1/COX/PGE2 inflammation pathway and CGRP expression in trigeminal ganglion. (A) The expression level of IL‐1β was examined after cells transfected with si‐Control or si‐IL‐1β lentivirus, detected by western blot. (B) The expression of miR‐34a‐5p in each group was detected by quantitative real‐time PCR. (C) The expression of NF‐κB p65 in each group was detected by quantitative real‐time PCR. (D) The expression of SIRT1 in each group was detected by quantitative real‐time PCR. (E) The expression of COX2 in each group was detected by quantitative real‐time PCR. (F) The expression of IL‐1β in each group was detected by quantitative real‐time PCR. (G) Apoptosis rate was detected by flow cytometry. Data are shown as means ± SD. *P < 0.05, versus the control group; ^#^ *P* < 0.05, versus the miRNA control + IL‐1β siRNA group; ^&^ *P* < 0.05, versus the miRNA34a‐5p + siRNA control group. Each experiment was repeated three times with similar results.

Then, we silenced the endogenous IL‐1β with or without overexpression of siR‐34a‐5p by using siRNA to detect the effect on the IL‐1β/NF‐κB p65/COX2 inflammation pathway. Western blot analysis showed that si‐IL‐1β#1 had the highest efficacy on down‐regulation of IL‐1β, and thus was then chosen for the following experiments (Fig. 4A). Quantitative real‐time PCR results showed that down‐regulation of IL‐1β significantly decreased the expression of IL‐1β/NF‐κB p65/COX2 and siR‐34a‐5p, but up‐regulated the expression of SIRT1. In addition, overexpression of miR‐34a‐5p attenuated the silencing of IL‐1β‐induced down‐regulation of IL‐1β/NF‐κB p65/COX2 and up‐regulation of SIRT1 (Fig. 4B–F).

Next, we up‐regulated miR‐34a‐5p by using lenti‐miR‐34a‐5p, and then we detected the expression levels of SIRT1, IL‐1β, miR‐34a‐5p, NF‐κB p65 and COX2 by quantitative real‐time PCR. The results showed that the expression of miR‐34‐5p was profoundly increased after the cells transduced with lenti‐miR‐34a‐5p. In addition, the expressions of IL‐1β, NF‐κB p65 and COX2 were also markedly elevated after overexpression of miR‐34a‐5p in trigeminal ganglion, whereas the expression of SIRT1 was markedly suppressed, suggesting that miR‐34a‐5p positively regulates the IL‐1β/NF‐κB p65/COX2 inflammation pathway but negatively regulates the expression of SIRT1 (Fig. 4B–F). Furthermore, in Fig. 4G, overexpression of miR‐34a induced apoptosis in primary rat trigeminal neurons.

### Up‐regulation of miR‐34a‐5p enhanced the IL‐1β/COX2/PGE2 inflammation pathway and CGRP release via down‐regulation of SIRT1 in trigeminal ganglion neurons

**Fig 5:**
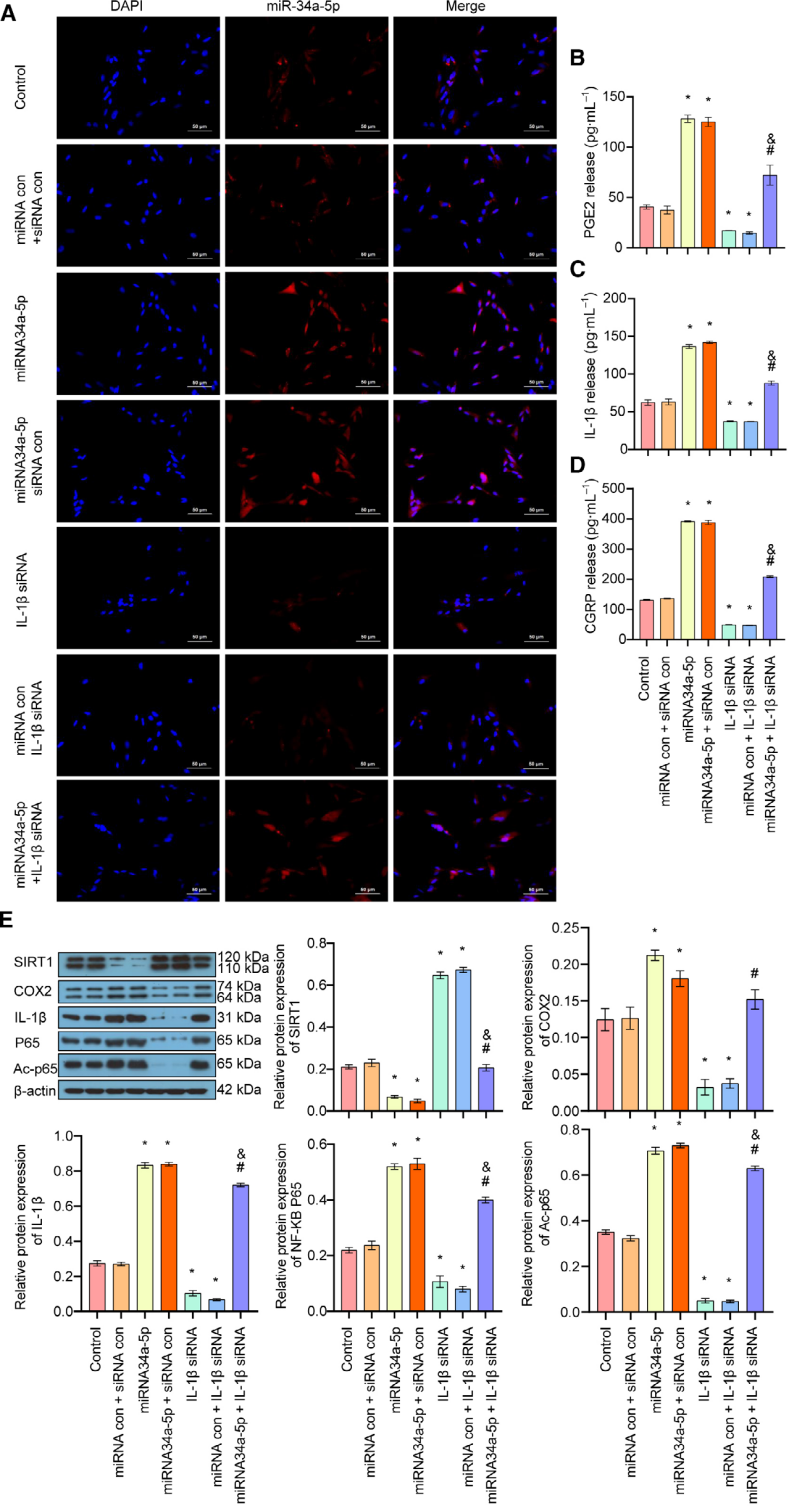
The effect of up‐regulation of miR‐34a‐5p/IL‐1β on the SIRT1/COX2/PGE2 inflammation pathway and CGRP release in trigeminal ganglion. (A) The expression and localization of miR‐34a‐5p in each group was detected by FISH assay (scale bar: 50 μm). (B) The release of PGE2 in each group was examined by ELISA. (C) The release of IL‐1β in each group was detected by ELISA. (D) The release of CGRP in each group was detected by ELISA. (E) The expressions of SIRT1, IL‐1β, p65, AC‐p65 and COX2 in each group were detected by western blot. Data are shown as means ± SD. *P < 0.05, versus the control group; ^#^ *P* < 0.05, versus the miRNA control + IL‐1β siRNA group; ^&^ *P* < 0.05, versus the miRNA34a‐5p + siRNA control group. Each experiment was repeated three times with similar results.

At last, we examined the expression of miR‐34a‐5p in trigeminal ganglion by FISH assay, and the results showed that overexpression of miR‐34a‐5p markedly increased the expression of miR‐34a‐5p in cells, whereas down‐regulation of IL‐1β decreased the expression of miR‐34a‐5p (Fig. 5A). Moreover, overexpression of miR‐34a‐5p alleviated silencing of IL‐1β‐induced reduction of miR34a‐5p (Fig. 5A). Furthermore, we examined the release of PGE2, IL‐1β and CGRP on overexpression of miR‐34a‐5p with or without silencing of IL‐1β. ELISA results demonstrated that the release of PGE2, IL‐1β and CGPR was remarkably elevated by miR‐34a‐5p lentiviral vector, whereas silencing of IL‐1β decreased the release of PGE2, IL‐1β and CGPR (Fig. 5B–D). Moreover, down‐regulation of IL‐1β attenuated miR‐34a‐5p overexpression‐induced increased release of PGE2, IL‐1β and CGPR (Fig. 5B–D). Western blot analysis in Fig. 5E showed that overexpression of miR‐34a‐5p significantly decreased the protein expressions of SIRT1. It enhanced the expression of IL‐1β, NF‐κB p65, AC‐p65 and COX2. However, silencing of IL‐1β increased the protein expression of SIRT1 and suppressed the expression of IL‐1β, NF‐κB p65 AC‐p65 and COX2. It also attenuated the miR‐34a‐5p‐induced up‐regulation of these proteins (Fig. 5E). Altogether, these results suggested that overexpression of miR‐34a‐5p enhanced the IL‐1β/COX2/PGE2 inflammation pathway and CGRP release in trigeminal ganglion via down‐regulation of SIRT1.

## Discussion

Migraine ranks among the top 10 causes of disability with a complex pathogenesis [20]. Currently, the origin of pain in migraine is still under debate, and the literature supports the view that headache attacks of migraine may involve nociceptive signals originating from intracranial and extracranial areas that are sensitive to pain, and that the signals propagate to the central trigeminal neurovascular neurons through peripheral nociceptors [2, 4]. Current therapies for treatment of migraine are only partially effective for some of the patients [21, 22]. Therefore, research has been focused on finding new biomarkers for migraine to further improve the therapeutic efficacy.

Recently, accumulative evidences show that miRNAs play an important role in the pathogenesis of migraine [11, 23, 24]. Altered expression of miRNA has been reported to be a potential biomarker for the headache disorder, including migraine [11, 23]. Among these miRNAs, miR‐34a‐5p was reported to be up‐regulated during migraine attacks, and miR‐34a‐5p has the potential to be used as a biomarker for prediction of therapeutic response in migraine [11, 23]. In addition, previous study showed that overexpression of miR‐34a enhances the IL‐1β expression [12, 25], whereas down‐regulation of miR‐34a induces the expression of SIRT1, reduces the activation of NF‐κB and inhibits the expressions of inflammatory factors, such as IL‐1β, IL‐6 and tumor necrosis factor‐α [13]. SIRT1 is an NAD^+^‐dependent protein deacetylase that has been shown to suppress the NF‐κB signaling pathway by directly deacetylating the p65 subunit of NF‐κB to inhibit the inflammatory responses mediated by downstream transcription factors [14]. Therefore, miR‐34a may increase the level of Ac‐p65 by inhibiting SIRT1 and activate IL‐1β transcription.

In this study, we used gain‐ and loss‐of function studies to investigate the role of miR‐34a‐5p on the regulation of the NF‐κB/IL‐1β inflammation pathway. Our results showed that down‐regulation of miR‐34a‐5p by its inhibitor decreased the expression levels of IL‐1β, NF‐κB p65 and AC‐p65 in both mRNA and protein levels, indicating the inhibition on inflammation and NF‐κB activation. In addition, miR‐34a‐5p inhibitor enhanced the expression of SIRT1. Moreover, silencing of SIRT1 by siRNA up‐regulated the expression of IL‐1β, NF‐κB p65 and AC‐p65 and attenuated the inhibition of miR‐34a‐5p‐induced down‐regulation of these proteins. Furthermore, a gain‐of‐function study using a lentiviral vector to overexpress miR‐34a‐5p results demonstrated that overexpression of miR‐34a‐5p up‐regulated the expression of IL‐1β, NF‐κB p65 and AC‐p65 but down‐regulated the expression of SIRT1. Silencing of the SIRT1 had the opposite effect and attenuated the overexpression of miR‐34a‐5p‐induced up‐regulation of IL‐1β, NF‐κB p65 and AC‐p65. These results are accordance with a previous report and for the first time suggest that miR‐34a‐5p increased the level of IL‐1β and NF‐κB p65 via down‐regulation of SIRT1 in trigeminal ganglion neurons.

PGE2 is a COX product, which is a crucial mediator of inflammatory pain sensitization [15, 16]. Previous studies demonstrated that COX2 can enhance the synthesis of PGE2 in the central nervous system, thereby promoting inflammatory pain and neuronal sensitization and elevating the degree of inflammatory pain [15, 17]. CGRP has long been shown to play an important role in the pathophysiology of migraine [5, 19]. Activation of the trigeminal nerve triggers the release of CGRP from perivascular nerve endings, and the serum CGRP will be significantly elevated during migraine attack [5, 19]. Therefore, our hypothesis was that overexpression of miR‐34a‐5p may inhibit the expression of SIRT1 and up‐regulation of IL‐1β/COX2/PEG2, thereby triggering the release of CGRP to induce the pain during migraine. In this study, we detected the expression of COX2 and the release of PEG2 and CGRP after down‐ or up‐regulation of miR‐34a‐5p. The results demonstrated that down‐regulation of miR‐34a‐5p decreased the expression of COX2, whereas up‐regulation of miR‐34a‐5p increased the expression of COX2. Moreover, silencing of SIRT1 attenuated the effect of inhibition of miR‐34a‐5p on COX2, whereas down‐regulation of IL‐1β alleviated the effect of overexpression of miR‐34a‐5p on COX2 expression. ELISA results showed that the release of IL‐1β, PEG2 and CGRP was decreased by inhibition of miR‐34a‐5p inhibitor and increased by overexpression of miR‐34a‐5p. Silencing of SIRT1 attenuated the inhibition effect of miR‐34a‐5p on the release of IL‐1β, PEG2 and CGRP. Down‐regulation of IL‐1β decreased the release of IL‐1β, PEG2 and CGRP, indicating that IL‐1β might induce the expression of COX2, which in turn promoted the expression and release of PEG2. miR‐34a‐5p is reported to play important roles in inducing different cell type apoptosis [26]. In this research, we found that overexpression of miR‐34a‐5p induced apoptosis in primary rat trigeminal neurons. We speculated that miR‐34a‐5p might activate the inflammatory pathway though inducing apoptosis. However, there are still limitations for this study. Previous research showed that IL‐1β might not be the direct target of miR‐34a [12]. Due to the constraint in investigation funding and time limitations, we will study the association between miR34a‐5p and IL‐1β in the future, and we hope to make contributions in the clinical practice in pain relief for patients.

## Conclusions

In summary, overexpression of miR‐34a‐5p inhibited the expression of SIRT1 and increased the expression or release of IL‐1β/COX2/PEG2, which further enhanced the release of CGRP, thereby triggering the pain during migraine onset. These results uncovered that miR‐34a‐5p has the potential to be explored as a potential therapeutic target for the treatment of migraine.

## Conflict of interest

The authors declare no conflict of interest.

## Author contributions

HZ guided the experiment, analyzed the results and wrote the manuscript. SH designed the whole experiment and organized the data. HZ, XZ, DZ, XJ, HJ, FZ and SH conducted the experiment.

## Data Availability

The datasets used and/or analyzed during this study are available from the corresponding author on reasonable request.
